# Supplementary material for: SARS-CoV-2 specific T cell responses are lower in children and increase with age and time after infection
Source: Nat Commun. 2021 Jul 29;12:4678. doi: 10.1038/s41467-021-24938-4 (PMC8322064; doi:10.1038/s41467-021-24938-4)
Supplement: Supplementary file 1 — Supplementary Information [file 41467_2021_24938_MOESM1_ESM.pdf]

Supplementary Table 1. T cell response in COVID-19 patients, trend and versus controls

		Trend days after symptom onset		Children vs Adults		COVID-19 cases vs Controls		
T cells		Estimate	P-value	Estimate	P-value	Median (case)	Median (negative)	P-value
CD4	Structural IFNγ^+ (total)	0.000012	0.920	-0.029	0.01	0.0479	0.00999	0.003
	ORF1ab IFNγ^+ (total)	0.00016	0.310	0.017	0.297	0.0235	0.0115	0.004
	Accessory IFNγ^+ (total)	-0.000037	0.256	-0.0043	0.153	0.0124	0.0134	0.350
	Structural TEM	0.0032	0.950	-3.2	0.493	88.7	89.5	0.552
	Structural TCM	-0.017	0.577	1.5	0.625	9.28	8.54	0.752
	Structural IFNγ^+TNF$^+$	-0.0000095	0.637	-6.00E-04	0.756	0.00436	0.000813	0.051
	Structural IFNγ^+TNF$^+$IL2$^+$	-0.000086	0.003	-0.0077	0.232	0.00342	0.000723	0.068
CD8	Structural IFNγ^+ (total)	0.00014	0.592	-0.094	0.009	0.0345	0.0395	0.884
	ORF1ab IFNγ^+ (total)	0.00052	0.596	-0.27	0.008	0.101	0.101	0.818
	Accessory IFNγ^+ (total)	-0.000029	0.084	-0.0021	0.184	0.00796	0.0157	0.156
	Structural TEM	-0.045	0.452	7.7	0.232	77.4	68.5	0.350
	Structural TCM	0.10	0.006	-2.1	0.546	10.6	20.6	0.022
	Structural IFNγ^+TNF$^+$	0.0000050	0.678	-0.0015	0.219	0.000595	0	0.036
	Structural IFNγ^+TNF$^+$IL2$^+$	0.00000031	0.845	-0.00041	0.276	0	0	0.315

* P value by Mann-Whitney test, significant values in red

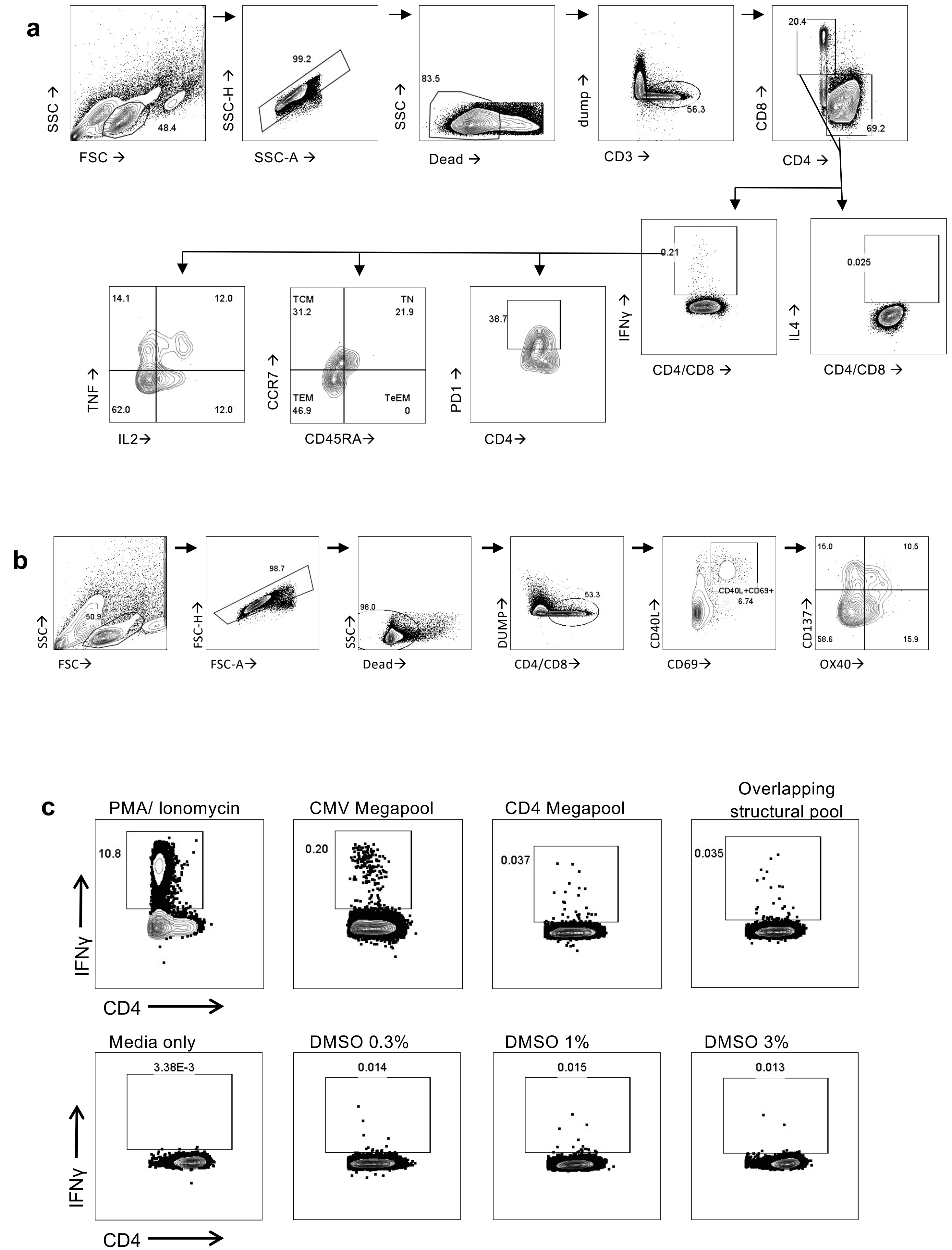

Supplementary Figure 1 – SARS-CoV-2 specific cells for cytokine production and phenotype by Flow cytometry. (a) The gating strategy for the characterisation of IFNγ and IL4 responses for $CD4^+$ and $CD8^+$ T cells for cytokine production (IL4, IFNγ, TNF, IL2), memory phenotype (CCR7, CD45RA), and exhaustion markers (PD1). (b) Gating strategy for detection of activation induced markers (CD40L, CD69, CD137 and OX40) on $CD4^+$ and $CD8^+$ T cells. (c) FACS plots showing $CD4^+$ IFNγ production to positive controls, PMA/Ionomycin, a CMV megapool and a CD4 specific megapool (from Grifoni *et al*. 2020), and negative controls with a range of DMSO concentrations.

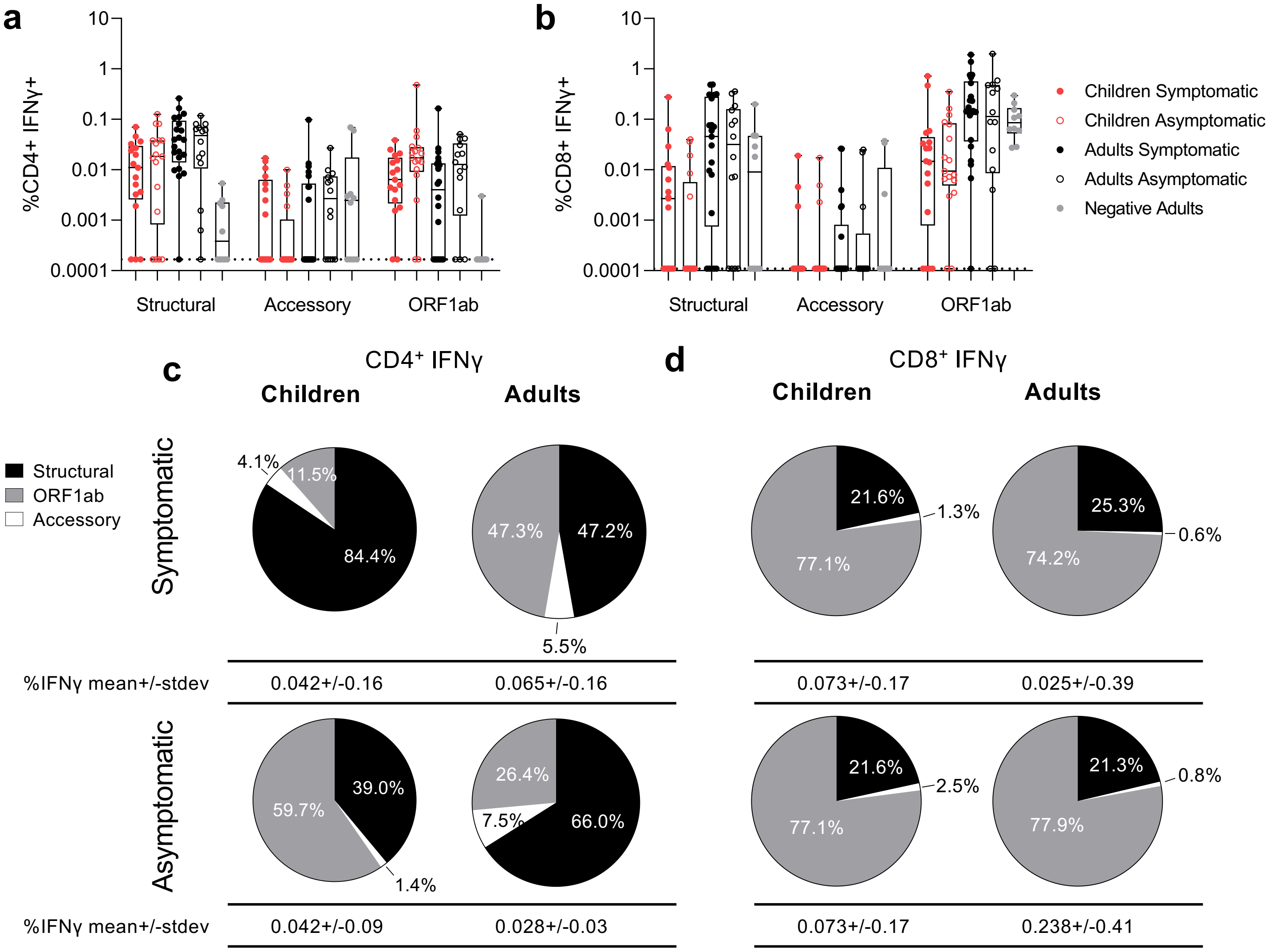

Supplementary Figure 2 - IFNγ CD4⁺ and CD8⁺ T cell responses are not different between symptomatic or asymptomatic SARS-CoV-2 infected children or adults. The SARS-CoV-2 CD4⁺ (a) or CD8⁺ (b) T cell responses of COVID-19 symptomatic children (n= 17, mean±stdev: 38.8±40.6 days), asymptomatic children (n= 17, 26.2±39.3 days), symptomatic adults (n= 22, 60.3±55.5 days), asymptomatic adults (n= 14, 32.8±32.3 days), from acute, convalescent and long-term memory time points (day 1 to 180 post symptom onset) and negative controls (n=10). Data represents the individual response with background subtracted, box and whisker plots median with upper and lower quartiles, minimum and maximum values. Multiple comparisons were performed using Kruskal-Wallis test for between-group comparison. Pie charts of total IFNγ⁺ CD4⁺ (c) and CD8⁺ (d) T cell SARS-CoV-2 responses with background subtracted and non-responders assigned a response of zero (from a, b).

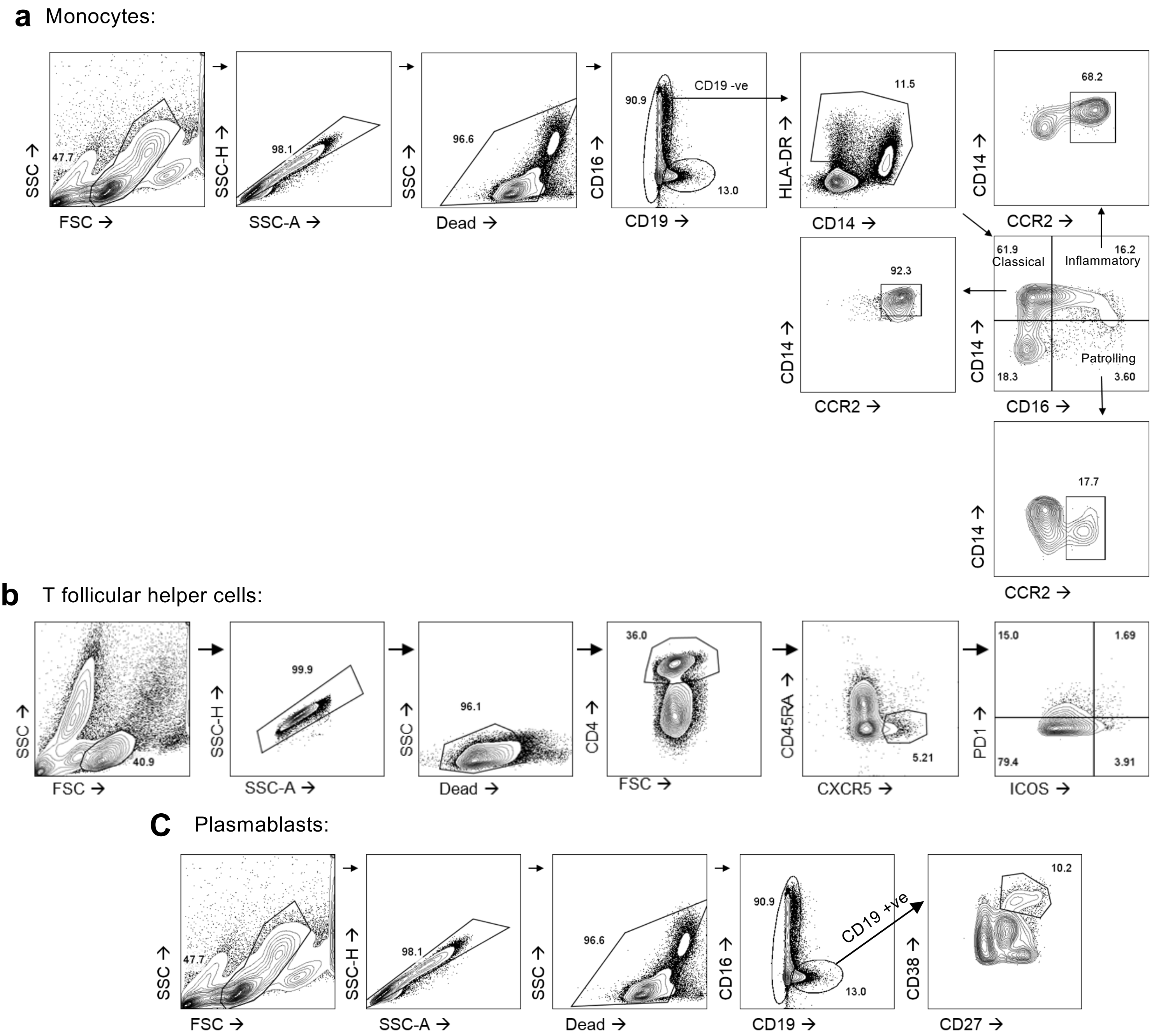

Supplementary Figure 3 – Cellular recruitment during acute infection of children shows increased Tfh responses. (a) The gating strategy for characterisation of total, classical, inflammatory and patrolling monocytes and their activation levels (by CCR2). Gating strategy for activated T follicular helper cells (b) and total plasmablasts (c).

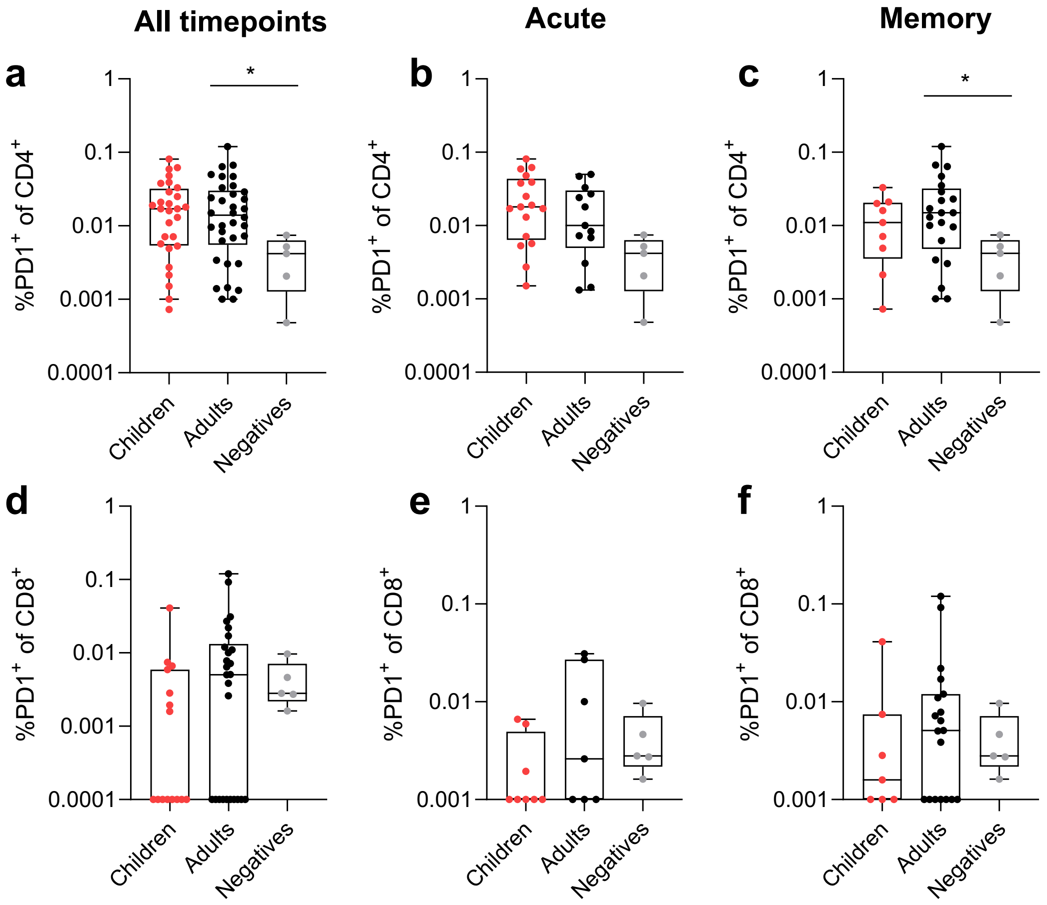

Supplementary Figure 4 – T cell exhaustion is equivalent in adult and children CD4 and CD8 T cells. $PD1^+$ $IFN\gamma^+$ $CD4^+$ (a-c) and $CD8^+$ (d-f) T cells in response to stimulation with structural pool at all time points (a, d) (day 1-180) in children (red), adults (black) and negative adults (grey) who are responders. Data separated into acute (b, e) (day<14 post symptom onset) and convalescent/memory (c, f) (day 15-180 post infection) time points. (a-f) Data is presented as individual data points with box and whiskers showing median, upper and lower quartiles and minimum and maximum values. Comparisons between infected children and adults, or infected adults and negative adults are carried out using Mann-Whitney (unpaired) test where $*p<0.05$, (a) $*p=0.0298$ (c) $*\ p=0.0393$.
