# Supplementary material for: SARS-CoV-2 specific T cell responses are lower in children and increase with age and time after infection
Source: Nat Commun. 2021 Jul 29;12:4678. doi: 10.1038/s41467-021-24938-4 (PMC8322064; doi:10.1038/s41467-021-24938-4)
Supplement: Supplementary file 3 — Reporting Summary [file 41467_2021_24938_MOESM3_ESM.pdf]

nature research

Corresponding author(s): Valkenburg

Last updated by author(s): Jun 23, 2021

Reporting Summary

Nature Research wishes to improve the reproducibility of the work that we publish. This form provides structure for consistency and transparency in reporting. For further information on Nature Research policies, see our Editorial Policies and the Editorial Policy Checklist.

Statistics

For all statistical analyses, confirm that the following items are present in the figure legend, table legend, main text, or Methods section.

n/a	Confirmed	
☐	☒	The exact sample size (*n*) for each experimental group/condition, given as a discrete number and unit of measurement
☐	☒	A statement on whether measurements were taken from distinct samples or whether the same sample was measured repeatedly
☐	☒	The statistical test(s) used AND whether they are one- or two-sided<br>*Only common tests should be described solely by name; describe more complex techniques in the Methods section.*
☒	☐	A description of all covariates tested
☐	☒	A description of any assumptions or corrections, such as tests of normality and adjustment for multiple comparisons
☐	☒	A full description of the statistical parameters including central tendency (e.g. means) or other basic estimates (e.g. regression coefficient) AND variation (e.g. standard deviation) or associated estimates of uncertainty (e.g. confidence intervals)
☐	☒	For null hypothesis testing, the test statistic (e.g. *F*, *t*, *r*) with confidence intervals, effect sizes, degrees of freedom and *P* value noted<br>*Give P values as exact values whenever suitable.*
☒	☐	For Bayesian analysis, information on the choice of priors and Markov chain Monte Carlo settings
☒	☐	For hierarchical and complex designs, identification of the appropriate level for tests and full reporting of outcomes
☐	☒	Estimates of effect sizes (e.g. Cohen's *d*, Pearson's *r*), indicating how they were calculated

Our web collection on statistics for biologists contains articles on many of the points above.

Software and code

Policy information about availability of computer code

Data collection	- Invitrogen AttuneNxT flow cytometer<br>- Wallac MicroBeta JET luminometer (PerkinElmer)
Data analysis	- FlowJo (Version 10)<br>-Graphpad Prism (Version 9)

For manuscripts utilizing custom algorithms or software that are central to the research but not yet described in published literature, software must be made available to editors and reviewers. We strongly encourage code deposition in a community repository (e.g. GitHub). See the Nature Research guidelines for submitting code & software for further information.

Data

Policy information about availability of data

All manuscripts must include a data availability statement. This statement should provide the following information, where applicable:

- Accession codes, unique identifiers, or web links for publicly available datasets
- A list of figures that have associated raw data
- A description of any restrictions on data availability

The protein, peptide sequences and data that support the findings of this study are available from the corresponding author upon reasonable request. The amino acid sequence of the peptide pools was based on βCoV/Hong Kong/VM20001061/2020 strain under accession code GenBank: MT547814.1 [https://www.ncbi.nlm.nih.gov/nuccore/MT547814]. Data from flow cytometry and ELISA IgG responses with background subtracted are indicated in all figures, and representative flow cytometry plots are shown.

Field-specific reporting

Please select the one below that is the best fit for your research. If you are not sure, read the appropriate sections before making your selection.

☒ Life sciences ☐ Behavioural & social sciences ☐ Ecological, evolutionary & environmental sciences

For a reference copy of the document with all sections, see nature.com/documents/nr-reporting-summary-flat.pdf

Life sciences study design

All studies must disclose on these points even when the disclosure is negative.

Sample size	We used opportunistic sampling of RT-PCR COVID-19 patients that were available in Hong Kong were recruited for this study. No sample size calculation was performed. An initial sample size of n=24 children, 45 adults SARS-CoV-2 patients. Multiple longitudinal samples were obtained from several donors. Follow up experiments used a further sample size of n=15 children and n=15 adult SARS-CoV-2 patients. n=15 children and n=25 adults age- and sex-matched uninfected controls was used in our study.
Data exclusions	One sample was excluded from data analysis due to a problem during acquisition by flow cytometry.
Replication	Experiments were successfully repeated at least twice on independent samples.
Randomization	Control samples were not randomized but selected based on matching age and gender to SARS-CoV-2 RT-PCR confirmed infected subjects samples age and gender.
Blinding	We were not blinded to subjects as recruitment was based on RT-PCR confirmed COVID-19 infection, and control samples selected based on age and gender matching.

Reporting for specific materials, systems and methods

We require information from authors about some types of materials, experimental systems and methods used in many studies. Here, indicate whether each material, system or method listed is relevant to your study. If you are not sure if a list item applies to your research, read the appropriate section before selecting a response.

Materials & experimental systems

n/a	Involved in the study	
☐	☒	Antibodies
☒	☐	Eukaryotic cell lines
☒	☐	Palaeontology and archaeology
☒	☐	Animals and other organisms
☐	☒	Human research participants
☒	☐	Clinical data
☒	☐	Dual use research of concern

Methods

n/a	Involved in the study	
☒	☐	ChIP-seq
☐	☒	Flow cytometry
☒	☐	MRI-based neuroimaging

Antibodies

Antibodies used	HRP Anti-Human IgG, Clone G18-145 (BD Pharmingen, Cat 555788) at 1:5,000 dilution<br>Zombie-NIR (cat # 423106, 1:1,000)<br>All antibodies from Biolegend catalogue and clone used<br>anti-human CD3-PE/Dazzle 594 (980006, UCHT1, 1:200), CD4-BV605 (317438, OKT4, 1:100), CD8-AlexaFluor700 (344724, SK1, 1:100), CCR7-PerCP/Cy5.5 (353220, G043H7, 1:20), PD-1-BV421 (367422, NAT105, 1:50), CD25-PE (302606, BC96, 1:100) and CD45RA-APC (983004, HI100, 1:200) and a dump channel containing CD19-BV510 (302242, HIB19, 1:100), CD56-BV510 (318340, HCD56, 1:100) and CD14-BV510 (301842, M5E2, 1:100). Cells were then permeabilised and fixed (BD Cytofix/cytoperm) and further stained for anti-human IFNg-FITC (502506, 4S.B3, 1:50), IL-2-PECy7 (500326, MQ1-17H12, 1:50), TNFa-BV711 (502940, MAb11, 1:50). L4-PE (500810, MP4-25D2, 1:50).<br>CD154-PE/Dazzle 594 (310840, 24-31, 1:100), OX40-PE (350004, ACT35, 1:100), CD137-BV711 (309832, 4B4-1, 1:50), CD69-FITC (310904, FN50, 1:100) and CXCR5-PE-Cy7 (145516, L138D7, 1:50), CD16-PE (980102, 3G8, 1:200), CD14-PerCPCy5.5 (301824, M5E2, 1:100), HLA-DR-BV605 (307640, L243, 1:75), CCR2-APC (357208, K035C2, 1:100), CD19-BV510 (302242, HIB19, 1:100), CD27-FITC (356404, M-T271, 1:50) and CD38-BV421 (303526, HIT2, 1:100). The Tfh panel contained: anti-human CD4-AlexaFluor700 (344622, SK3, 1:100), CXCR5-PerCPCy5.5 (356910, J252D4, 1:50), CD45RA-FITC (983002, HI100, 1:200), PD-1-BV711 (329928, EH12.2H7, 1:50) and ICOS-PE (313508, C398.4A, 1:50)
Validation	Product specification sheets for each antibody describe from the company the expected cell proportion based on cell staining. All antibodies were titrated by us prior to use on patient PBMC samples from uninfected healthy donors to determine optimal staining concentrations, and activation markers assessed after PMA/ionomycin stimulation. Single colour florescence controls are acquired for each antibody at the time of data acquisition to ensure antibodies in use are working well.

Human research participants

Policy information about studies involving human research participants

Population characteristics	RT-PCR-confirmed infected patients were recruited to participate in immune studies of COVID-19. All patients and the parents of children provided informed consent. RT-PCR+ COVID-19 patients in Hong Kong were recruited for this study through their consulting physician. An initial sample size of infected n=24 children (mean stdev: 8.1±3.9, range: 1.92 (23 months)-13 years), 45 adults (mean±stdev: 43.1±13.7, range: 20-65 years) SARS-CoV-2 patients and uninfected n=15 children (10.3±3.2, 2-14 years) and 25 adults (37.6±13.0, 19-57 years) age- and sex-matched controls were used in our study. The details of their age, gender and patient symptom severity is given in Table 1.
Recruitment	RT-PCR confirmed COVID-19 infection were enrolled during clinical care in hospitals in Hong Kong (China, SAR) and all of them provided informed consent. Their blood was collected at various time-point after disease onset, including hospital admission and discharge and long term follow up with clinicians, and there was no bias to the recruitment or collection. Samples were selected from a large biobank of patients for time point similarity between infected adults and children, and age matching for negative controls.
Ethics oversight	The study was approved by the institutional review board of the respective hospitals, viz. Kowloon West Cluster (KW/EX-20-039 (144-27)), Kowloon Central/Kowloon East cluster (KC/KE-20-0154/ER2) and HKU/HA Hong Kong West Cluster (UW 20-273, UW20-169), Joint Chinese University of Hong Kong-New Territories East Cluster Clinical Research Ethics Committee (CREC 2020.229). All of patients, and children and their parents provided informed consent. The collection of SARS-CoV-2 seronegative adult negative control blood donors (37.6±13.0, 19-57 years) was approved by the Institutional Review Board of The Hong Kong University and the Hong Kong Island West Cluster of Hospitals (UW16-254). SARS-CoV-2 seronegative children's control blood donors (n=15) were recruited from immunocompetent children from renal, endocrine and blood clinics (10.3±3.2, 2-14 years) who were donating blood for non-infection related purposes. Informed consent was given by patients and parents and the collection of these samples was approved by HKU/HA Hong Kong West Cluster Hospitals (UW 20-273, UW20-169).

Note that full information on the approval of the study protocol must also be provided in the manuscript.

Flow Cytometry

Plots

Confirm that:

☒ The axis labels state the marker and fluorochrome used (e.g. CD4-FITC).

☒ The axis scales are clearly visible. Include numbers along axes only for bottom left plot of group (a 'group' is an analysis of identical markers).

☒ All plots are contour plots with outliers or pseudocolor plots.

☒ A numerical value for number of cells or percentage (with statistics) is provided.

Methodology

Sample preparation	PBMC samples were isolated from heparinised blood by density gradient centrifugation and frozen in FBS/DMSO. Samples were thawed and washed twice with media, and stimulated with overlapping peptide pools.
Instrument	AttuneNxT, Invitrogen
Software	FlowJo (version 10)
Cell population abundance	At least 200,000 live cell events were acquired.
Gating strategy	Gating strategy provided as supplementary figures (Supplementary figures 1 and 3).

☒ Tick this box to confirm that a figure exemplifying the gating strategy is provided in the Supplementary Information.
